# Admission of kidney patients to a closed staff nephrology department results in a better short-term survival

**DOI:** 10.1371/journal.pone.0279172

**Published:** 2023-03-07

**Authors:** Lihi Schwartz, Omer Rosenshtok, Leah Shalev, Ella Schneider, Anna Basok, Marina Vorobiov, Elvira Romanjuk, Boris Rogachev, Ismail El-Sayed, Lina Schwartz, Idan Menashe, Ohad Regev, Yosef S. Haviv

**Affiliations:** 1 Faculty of Health Sciences, Goldman Medical School, Ben-Gurion University of the Negev, Be’er Sheva, Israel; 2 Department of Nephrology, Soroka University Medical Centre, Beer-Sheva, Israel; 3 The Faculty of Health Sciences, Department of Public Health, Ben-Gurion University of the Negev, Beer-Sheva, Israel; 4 Faculty of Health Sciences, Ben-Gurion University, Beer-Sheva, Israel; Faculty of Medicine, Saint-Joseph University, LEBANON

## Abstract

**Background:**

The outcome of patients with chronic kidney disease (CKD) and acute kidney injury (AKI) is often dismal and measures to ameliorate their course are scarce. When admitted to the hospital, kidney patients are often hospitalized in general Medicine wards rather than in a specialized Nephrology department. In the current study, we compared the outcome of two cohorts of kidney patients (CKD and AKI) admitted either to general open-staff (with rotating physicians) Medicine wards or to a closed-staff (non-rotating Nephrologists) Nephrology ward.

**Methods:**

In this population-based retrospective cohort study, we enrolled 352 CKD patients and 382 AKI patients admitted to either Nephrology or General Medicine wards. Short-term (< = 90 days) and long-term (>90 days) outcomes were recorded for survival, renal outcomes, cardiovascular outcomes, and dialysis complications. Multivariate analysis was performed using logistic regression and negative binomial regression adjusting to potential sociodemographic confounders as well as to a propensity score based on the association of all medical background variables to the admitted ward, to mitigate the potential admittance bias to each ward.

**Results:**

One hundred and seventy-one CKD patients (48.6%) were admitted to the Nephrology ward and 181 (51.4%) were admitted to general Medicine wards. For AKI, 180 (47.1%) and 202 (52.9%) were admitted to Nephrology and general Medicine wards, respectively. Baseline age, comorbidities and the degree of renal dysfunction differed between the groups. Using propensity score analysis, a significantly reduced mortality rate was observed for kidney patients admitted to the Nephrology ward vs. general Medicine in short term mortality (but not long-term mortality) among both CKD patients admitted (OR = 0.28, CI = 0.14–0.58, p = 0.001), and AKI patients (or = 0.25, CI = 0.12–0.48, p< 0.001). Nephrology ward admission resulted in higher rates of renal replacement therapy (RRT), both during the first hospitalization and thereafter.

**Conclusions:**

Thus, a simple measure of admission to a specialized Nephrology department may improve kidney patient outcome, thereby potentially affecting future health care planning.

## Introduction

A number of studies have shown that it is remarkably difficult to ameliorate the outcome of kidney patients using medical or technical measures for both acute kidney injury (AKI) and end stage renal disease (ESRD). In contrast, the outcome of CKD can be improved with novel drugs. On the other hand, administrative steps facilitating kidney patient-Nephrologist interaction may improve their outcome, e.g. earlier out-patient referral to a Nephrologist can reduce mortality and hospitalizations [1]. Thus, specialist involvement may be beneficial in the out-patient Nephrology setting However, in the context of in-patients, whether admission to a specialized Nephrology department improves survival is yet to be determined.

Health care utilization among adult CKD patients is high and 47% of the patients are hospitalized at least once per year [2,3]. When kidney patients are hospitalized their outcome is worse than patients with intact renal function [4]. Kidney patients are often hospitalized in general Medicine wards where Nephrologist consultation may be requested. These patients may be regarded as ‘outliers’ of the Nephrology ward with a substantially lower degree of specialist involvement.

Outliers in general may have an increased length of hospitalization [5], as shown by a study from another field (Neurology) that found a significantly shorter median length of stay in a specialist unit compared to the general wards (9 days vs 13 days respectively) [6]. In the field of Nephrology, Fagugli et al [7] investigated the outcome of patients with acute kidney injury (AKI) requiring dialysis who were admitted to either a Nephrology ward or to general medical wards. The study showed reduced in-hospital mortality in the Nephrology ward (20% versus 52%), thereby suggesting that for the most severe AKI patients requiring dialysis, specialty care may result in better outcomes. Other studies demonstrated that early Nephrologist involvement in patients with AKI may reduce the risk of further decrease in kidney function [8]. Moreover, delayed Nephrology consultation was associated with increased dialysis dependence rates in critically ill AKI patients on hospital discharge [9].

In the current in-patient study, we retrospectively examined whether the outcome of hospitalized kidney patients, i.e. AKI (AKIN classification stages 1–3) not requiring dialysis and CKD (stages G3-G5) patients, was improved following admission to a closed-staff Nephrology ward (see below classification). To the best of our knowledge these patient populations have not been examined in this regard previously.

## Methods

### Study design

This was a population-based retrospective cohort study comparing two cohorts of kidney patients (either AKI or CKD patients) admitted to general Medicine wards with Nephrology consultation vs. care in a closed-staff Nephrology ward. Short-term (< = 90 days) and long-term (>90 days) outcomes were recorded for mortality, renal outcome (RRT (dialysis or kidney transplantation)) and AV shunt surgery, composite dialysis complication score (CDCs), CREDENCE composite outcome [see below]), and cardiovascular outcomes [MACE, see below]. Of note, AV shunt surgery differs from the other outcomes in predicting a better prognosis in dialysis patients [10].

### Setting

Soroka University Medical Center is the 4^th^ largest hospital in Israel and the only one in the Negev district providing medical services to ~ 1 million residents. Because all the kidney patients in the Negev district are referred to Soroka University Medical Center, admissions to Soroka hospital were considered to reflect all hospitalization events.

The Medicine wards are based on an open-staff structure, i.e., both the attending senior physician in the medical wards and the consulting Nephrologist are rotating, the former monthly and the latter daily. In contrast, in the closed-staff Nephrology department the staff is unchanged and board-certified in Nephrology. Daily morning meetings of 6–8 Nephrologists are conducted to guide patient care. The Nephrology floor comprises a 12-bed ward dedicated entirely to kidney in-patients, in addition to peritoneal dialysis outpatient unit, hemodialysis outpatient and in-patient unit, and a kidney transplantation service. The medical staff comprises 7 board-certified Nephrologists and one resident. The nurses all passed a 1-yr Nephrology and dialysis nursing course. In-house dietitian and social worker guide the relevant aspects of therapy.

### Study participants and data sources

The two kidney patient cohorts were defined as AKI or CKD. All patients were adults (>18 years) with renal dysfunction admitted either to the Nephrology Ward or to the General Medicine wards (in the latter only patients with Nephrology consultation were included). The dates of admission were from 21 July 2016 through 31 December 2018 (exclusion criteria common to both cohorts were absence of Nephrologist consultation, need for urgent dialysis on admission, admission to ICU or surgery and ESRD (on chronic dialysis or with a kidney transplant) [S1 Table]. The specific AKI study exclusion criterion was serum creatinine rise below 50% compared to baseline. The latter was calculated as the mean of the available serum creatinine levels measured during the last year before admission.

Additional specific CKD study exclusion criteria were eGFR >60 ml/min. The data collection ended on 31.12.2019; thus, all patients have had at least one year of follow-up.For each patient we calculated the relevant AKIN/CKD KDIGO scores based on their creatinine level and rekevant demographic data. Because the serum creatinine alone does not accurately reflect the kidney function, these data were convertedninto the AKIN stage /CKD as the unit of analysis. The AKIN classification of AKI was used; AKI patients were classified into 3 stages [1.5-fold≤Serum creatinine (Scr) ≤2-fold, 2-fold <Scr≤ 3-fold, Scr> 3-fold] [11]. For CKD, The KDIGO classification was used; [G3a (45≤eGFR<60), G3b (30≤eGFR<45), G4 (15≤eGFR<30) and G5nd (eGFR<15, G5 CKD patients not receiving RRT) [12].

### Data collection

The study was based on two computerized datasets: a Nephrology consultation database, which consists of records of hospitalized patients, from all the hospital wards requesting Nephrology consultation. The second is Soroka’s Chameleon electronic medical records database, which comprises records of all patients treated in Soroka hospital. Based on previous power calculations, two-thirds of the patients were randomly selected using an arbitrary digit of their ID number, as reported before [13]. The study was investigator-initiated and was approved by the Soroka University Medical Center institutional review board (IRB). All diagnoses were classified by the international classification of disease (ICD-9).

### Statistical analysis

For each kidney patient cohort (AKI/CKD), the sociodemographic and medical characteristics of Nephrology and General Ward patients were assessed using appropriate univariate statistics. Next, we assessed the association between admission type and clinical outcomes using appropriate univariate statistics. Categorical variables were assessed using Chi-Square test. Continuous variables were assessed using either T-test (for normal distribution) or Mann-Whitney test (in all other cases).

To assess the independent association between admission type and clinical outcomes, we conducted a multivariate analysis using either logistic regression (for dichotomous variables) or negative binomial regression (for counting variables) adjusted for the sociodemographic variables, which showed significant association with ward type (age, ethnicity, and number of children). In addition, to mitigate the potential admittance bias to each ward, a propensity score (PS) was created using a logistic regression assessing the effect of all medical background variables on the hospitalization ward as a dependent variable. The resulting PS was added as an independent variable to all the multivariate regression models [14]. Details concerning the specific statistical tests conducted for each variable can be seen at the footnote of each table. All analyses were conducted using SPSS Statistics V. 25 and R software. A two-sided test significance level of 0.05 was used throughout the entire study.

The association between admission type and outcome was studied for the following parameters: long- and short-term all-cause mortality,4-point MACE (major adverse cardiac event: nonfatal stroke, nonfatal MI, congestive heart failure (CHF), cardiovascular death), need for dialysis during the first hospitalization, need for chronic RRT (measured as RRT after discharge from first hospitalization), recurrent hospitalization and AV shunt surgery. For long-term Nephrology outcome, we used the CREDENCE composite CKD progression score index, comprising either ESRD, doubling of the serum creatinine level, renal or cardiovascular death [15]. To assess a specific dialysis quality index, we also tested a composite dialysis complication score (CDCs), comprising any of the following: CLABSI (catheter-induced bacteremia), pulmonary edema, hyperkalemia requiring urgent hemodialysis, need for any acute dialysis during first hospitalization and all-cause mortality.

## Results

### Baseline characteristics

Table 1 depicts the sociodemographic and comorbidities of kidney patients admitted to the Nephrology ward or to general Medicine wards (of whom only patients with Nephrology consultation were included). The impact of the admitting department on the outcome of kidney patients was studied for 2 groups, i.e. AKI and CKD. Only when significant for both AKI and CKD, the difference between the admitting departments deemed to reflect clinically meaningful difference. Thus, at baseline, age, and the prevalence of cardiovascular disease (composite coronary vascular disease, peripheral vascular disease, acute coronary syndrome, cerebrovascular event), and congestive heart failure (CHF), were all significantly higher in kidney patients admitted to Medicine wards (Table 1). On the other hand, patients admitted to the Nephrology department manifested a more advanced stage of either AKI or CKD (Table 1). To address a potential admittance bias, a propensity score analysis was performed in addition to standard multivariate analysis (adjusted OR, Table 3).

**Table 1:** Clinical and sociodemographic characteristics of patients.

Variable		Study Group ^a^		Nephrology Department	General Department		Pv
**Sociodemographic Background**							
Age, Years	Mean±SD	AKI		63.6 ±19.6	69.4 ±13.9		**<0.001** ^**b**^
		CKD		66.2±16.2	71.31±13.1		**0.001** ^**b**^
Ethnicity (Jewish)	No. (%)	AKI		144 (82.3)	170 (85.4)		0.409 ^c^
		CKD		131(77.5)	158(87.8)		**0.011** ^**c**^
Sex (male)	No. (%)	AKI		96 (53.3)	119 (58.9)		0.273^c^
		CKD		108(63.2)	107(59.1)		0.437^c^
Family Status (Married)	No. (%)	AKI		124 (61.7)	98 (55.4)		0.213^c^
		CKD		105(62.1)	118(65.2)		0.551^c^
Number of Children	Median (IQR)	AKI		2(1–4)	3(2–5)		**0.007** ^**d**^
		CKD		3(1–5)	3(2–6)		0.085^d^
**Medical Background**							
Diabetes Mellitus	No. (%)	AKI		83 (46.1)	109 (54.0)		0.126^c^
		CKD		95(55.6)	112(61.9)		0.228^c^
Cardiovascular Disease	No. (%)	AKI		71 (39.4)	105 (52.0)		**0.014** ^**c**^
		CKD		84(49.1)	116(64.1)		**0.005** ^**c**^
Heart Failure	No. (%)	AKI		34 (18.9)	65 (32.2)		**0.003** ^**c**^
		CKD		40(23.4)	75(41.4)		**<0.001** ^**c**^
Hypertension	No. (%)	AKI		132 (73.3)	155 (76.7)		0.443^c^
		CKD		147(86)	151(83.4)		0.509^c^
Malignancy	No. (%)	AKI		20 (11.1)	41 (20.3)		**0.014** ^**c**^
		CKD		21(12.3)	30(16.6)		0.253^c^
Hemoglobin Levels	Mean±SD	AKI		10.93 ± 2.40	10.83 ± 2.28		0.704 ^b^
		CKD		10.49±2.00	10.50±2.18		0.954^b^
Albumin Levels	Mean±SD	AKI		3.11 ± 0.70	3.22 ± 0.88		0.244 ^b^
		CKD		3.21±0.62	3.20±0.87		**0.009** ^**b**^
Staging	AKIN No. (%)	AKI	Stage 1	43 (24.0)	78 (38.6)		**<0.001** ^**c**^
			Stage 2	44 (24.6)	67 (33.2)		
			Stage 3	92 (51.4)	57 (28.2)		
	KDIGO No. (%)	CKD	G3A	27 (15.9)	40 (22.2)		**0.006** ^**c**^
			G3B	46 (27.1)	62 (34.4)		
			G4	75 (44.1)	71 (39.4)		
			G5	22 (12.9)	7 (3.9)		

Note: Boldface type indicates p<0.05.

AKI = Acute kidney injury, CKD = chronic kidney disease; RRT = Renal replacement therapy; AKIN = Acute Kidney Injury Network; KDIGO = Kidney Disease Improving Global Outcomes; IQR = Interquartile range; SD = Standard deviation.

^a^ AKI: Nephrology N = 180, General N = 202; CKD: Nephrology N = 171, General N = 181.

^b^ T-Test, ^c^ Chi Square, ^d^ Mann-Whitney.

### Mortality

In univariate analysis, a significantly higher rate of short-term all-cause mortality was found among the two groups of kidney patients admitted to the open-staff Medicine wards compared to the closed-staff Nephrology ward (Table 2).

**Table 2:** Association between department type and clinical outcomes, univariate analysis.

Variable		Study Group ^a^	Nephrology Department	General Department	Pv
Short Term all-cause mortality (< = 90 days)	No. (%)	AKI	24 (13.3)	85 (42.1)	**<0.001** ^**c**^
		CKD	19(11.1)	71(39.2)	**<0.001** ^**c**^
Long Term all-cause mortality (>90 days)^b^	No. (%)	AKI	33 (18.3)	47 (23.3)	0.237 ^**c**^
		CKD	36(21.1)	51(28.2)	0.121 ^**c**^
RRT in first hospitalization	No. (%)	AKI	53 (29.4)	29 (14.4)	**<0.001** ^**c**^
		CKD	43(25.1)	24(13.3)	**0.005** ^**c**^
RRT After first hospitalization	No. (%)	AKI	45 (25.0)	24 (11.9)	**<0.001** ^**c**^
		CKD	89(52.0)	36(19.9)	**<0.001** ^**c**^
AV Shunt Surgery	No. (%)	AKI	14 (7.8)	6 (3.0)	**0.035** ^**c**^
		CKD	34(19.9)	11(6.1)	**<0.001** ^**c**^
Number of Recurrent Hospitalizations	Median (IQR)	AKI	1 (0–4)	1 (0–3)	0.079^d^
		CKD	3(1–5)	1(0–3.5)	**0.001** ^**d**^
Short Term CDCs	Median (IQR)	AKI	0 (0–1)	1 (0–1)	**0.043** ^**d**^
		CKD	1(0–1)	1(0–1)	0.441^d^
Long Term CDCs	Median (IQR)	AKI	0 (0–1)	0 (0–1)	0.917 ^d^
		CKD	0 (0–1)	0 (0–1)	0.162 ^d^
Long Term CREDENCE	No. (%)	AKI	47 (26.1)	48 (23.8)	0.596 ^**c**^
		CKD	72(42.1)	58(32.0)	0.051 ^**c**^
MACE	No. (%)	AKI	42 (23.3)	55 (27.2)	0.596 ^**c**^
		CKD	51(29.8)	71(39.2)	0.064 ^**c**^

Note: Boldface type indicates p<0.05.

AKI = Acute kidney injury, CKD = chronic kidney disease; RRT = Renal replacement therapy; CDCs = Composite dialysis complication score; CREDENCE = The Computed Tomographic Evaluation of Atherosclerotic Determinants of Myocardial Ischemia; AV Shunt Surgery = Arteriovenous (AV) Shunt Surgery;IQR = Interquartile range.

Short term: < = 90 days; Long term: >90 days.

^a^ AKI: Nephrology N = 180, General N = 202; CKD: Nephrology N = 171, General N = 181.

^b^ Nephrology Department: N = 152, General Department: N = 110.

^c^ Chi Square, ^d^ Mann-Whitney.

Next, univariate analysis (Table 3) showed a significantly lower mortality rate for kidney patients admitted to Nephrology floor for both CKD (OR = 0.19, CI = 0.11–0.34, p<0.001) and AKI (OR = 0.21, CI = 0.13–0.35, p<0.001).

**Table 3:** Association between department type and clinical outcomes univariate analysis multivariate analysis.

Variable	Study Group	Department	Odds Ratio (OR)	95% CI	Pv	Adjusted Odds Ratio (aOR)^c^	95% CI	Pv
Short Term all-cause mortality (< = 90 days) ^a^	AKI	General	REF		**<0.001**	REF		**<0.001**
		Nephrology	0.21	0.13–0.35		0.25	0.12–0.48	
	CKD	General	REF		**<0.001**	REF		**0.001**
		Nephrology	0.19	0.11–0.34		0.28	0.14–0.58	
Long Term all-cause mortality (>90 days)^a^	AKI	General	REF		0.238	REF		0.979
		Nephrology	0.74	0.45–1.22		0.99	0.52–1.88	
	CKD	General	REF		0.112	REF		0.310
		Nephrology	0.68	0.42–1.11		0.71	0.37–1.37	
RRT in first hospitalization ^a^	AKI	General	REF		**<0.001**	REF		**0.003**
		Nephrology	2.49	1.50–4.14		2.57	1.39–4.75	
	CKD	General	REF		**0.005**	REF		**0.003**
		Nephrology	2.20	1.27–3.81		2.81	1.42–5.56	
RRT after first hospitalization ^a^	AKI	General	REF		**0.001**	REF		**0.038**
		Nephrology	2.47	1.44–4.26		2.06	1.04–4.06	
	CKD	General	REF		**<0.001**	REF		**0.004**
		Nephrology	4.37	2.73–7.01		2.50	1.35–4.65	
AV Shunt Surgery ^a^	AKI	General	REF		**0.042**	REF		0.089
		Nephrology	2.76	1.04–7.33		2.94	0.85–10.19	
	CKD	General	REF		**<0.001**	REF		**0.016**
		Nephrology	3.81	1.86–7.70		3.36	1.25–8.99	
Number of Recurrent Hospitalizations ^b^	AKI	General	REF		**0.040**	REF		**0.001**
		Nephrology	1.29	1.01–1.64		1.66	1.22–2.25	
	CKD	General	REF		**0.004**	REF		**0.016**
		Nephrology	1.42	1.12–1.81		1.51	1.08–2.10	
Short Term CDCs ^b^	AKI	General	REF			REF		
		Nephrology	0.87	0.64–1.20	0.402	1.10	0.74–1.64	0.646
	CKD	General	REF			REF		
		Nephrology	0.97	0.70–1.33	0.831	1.15	0.76–1.75	0.515
Long Term CDCs ^b^	AKI	General	REF			REF		
		Nephrology	0.97	0.67–1.40	0.867	1.30	0.82–2.07	0.272
	CKD	General	REF			REF		
		Nephrology	1.12	0.81–1.56	0.490	1.12	0.72–1.74	0.617
Long Term CREDENCE ^a^	AKI	General	REF			REF		
		Nephrology	1.13	0.71–1.80	0.596	1.42	0.77–2.62	0.261
	CKD	General	REF			REF		
		Nephrology	1.54	0.99–2.38	0.051	1.37	0.76–2.47	0.292
MACE ^a^	AKI	General	REF			REF		
		Nephrology	0.81	0.51–1.29	0.383	1.66	0.88–3.12	0.116
	CKD	General	REF			REF		
		Nephrology	0.66	0.42–1.03	0.065	1.16	0.64–2.10	0.615

Note: Boldface type indicates p<0.05AKI = Acute kidney injury, CKD = chronic kidney disease; RRT = Renal replacement therapy; CDCs = Composite dialysis complication score; CREDENCE = The Computed Tomographic Evaluation of Atherosclerotic Determinants of Myocardial Ischemia; AV Shunt Surgery = Arteriovenous (AV) Shunt Surgery; REF = Reference.

Short term: < = 90 days; Long term: >90 days.

Propensity score based on all medical background variables.

^a^ Logistic Regression.

^b^ Negative Binomial Regression.

^c^ Adjusted for propensity score, age, race, and number of children.

Next, using multivariate analysis, adjusted to potential confounders as well as to the propensity score analysis, the Nephrology ward related relative reduction rate in short term mortality was 72% and 75%, for CKD and AKI patients respectively (CKD: OR = 0.28, CI = 0.14–0.58, p = 0.001; AKI: OR = 0.25, CI = 0.12–0.48, p<0.001). However, the long-term all-cause mortality was not affected by the type of admitting department (Table 3). Remarkably, the propensity score analysis reiterated the protective effect of Nephrology ward admission, relevant for both kidney patient populations.

### Intermediate outcomes

To evaluate intermediate outcomes, we next tested for composite dialysis complication score (CDCs), CREDENCE, MACE and RRT.

Although univariate analysis initially suggested that in AKI patients there could be less acute complications (short term CDCs) for patients admitted to Nephrology (Table 2), further multivariate analyses indicated that this score did not differ significantly between the Nephrology and Medicine departments (Table 3). Similarly, univariate analysis initially suggested that CKD patients had borderline higher long term CREDENCE score and lower MACE score for patients admitted to Nephrology (Table 2). However, in multivariate analysis (Table 3), these differences were no longer significant.

Admission to Nephrology was associated with higher rates of renal replacement therapy (RRT) both in and after the first hospitalization, and consequently AV shunt surgery, observed in all kidney cohorts (Table 3).

## Discussion

In this retrospective cohort study, we found that admission to a closed-staff Nephrology ward was associated in two groups of kidney patients with reduced short-term mortality. These findings for kidney patients are consistent with the benefits of specialized care units in other fields [7–9,16,17].

Fagugli et al [7] have demonstrated the value of a closed-staff Nephrology department in a selected group of AKI patients requiring acute dialysis. In the current study, we found that the value of closed-staff Nephrology department care may further extend to hospitalization of two major kidney patient groups, i.e., AKI and CKD stage 3A-5.

The present study reveals that for these two groups of kidney patients, short-term all-cause mortality rate was significantly lower in the Nephrology department. While the mortality difference in the range of 72–75% in favor of Nephrology floor care is very high, we acknowledge it may be partially exaggerated by admittance bias. In contrast, the mortality risk was not totally skewed in favor of the Nephrology department as more severe baseline renal dysfunction, known to predict mortality [18], was observed in patients admitted to Nephrology in both groups. We thus employed a propensity score analysis, adjusted to comorbidities and demographic parameters. Since a randomized controlled trial of kidney patient admission is unlikely due to ethical considerations, this limited study may still be of importance in regard to the structure of Department of Medicine and its sub-specialties. The possibility that early kidney patient transfer to an empowered Nephrology Department may substantially increase short term survival cannot be ruled out.

The relative reduction in the mortality rate may be possibly explained by several conjectures. Nephrology ward is a specialized department managed 24/7 by the same group of Nephrologists. In contrast, rotating attending Nephrologist consultation is requested by the general Medicine wards every few days. Furthermore, the patient to staff ratios in the Nephrology floor are lower, allowing more attention to patients. Third, facilities such as dialysis unit and transplantation clinic are part of the Nephrology Department as are specialist dietitian, transplantation nurse and social worker. It thus appears that a major factor affecting kidney patient short term survival is the human factor where a trained, closed-staff Nephrology team may improve AKI and CKD patient survival. This structure may also account for our finding of higher rate of RRT and AV shunt surgery in AKI and CKD patients admitted to Nephrology department.

In contrast to short-term mortality, long term all-cause mortality was not associated with the ward type. The following factors may account for this observation. First, in the AKI cohort, long-term mortality may have been underestimated. In 16 studies AKI was associated with increased long-term mortality (up to 83% 5-yr mortality risk.) [16]. Because our maximum follow-up time was 2.4 years, the long-term effect of Nephrology department on AKI and CKD care may only be realized after a longer period. Second, our finding that admission to the Nephrology Department was beneficial only for short-term mortality may in fact reflect the critical need for expertise in the management of kidney patients, whereas long-term mortality is multi-factorial involving general practitioners, pharmacists, nurses, and dietitians who are not always acquainted with the subtleties of CKD care. In this regard, outpatient Nephrology clinic visit were not accounted for after discharge.

The benefit of in-patient Nephrology care was previously reported primarily for AKI patients. Meier et al found that hospital-acquired AKI patients who had been referred early (within 5 days after development of AKI) to a Nephrologist were at lower risk for in-hospital morbidity and mortality compared with non-Nephrologist referral and late (> 5 days) Nephrology referral [17]. A similar difference in short-term mortality in a subgroup of 296 non critically ill AKI patients requiring acute dialysis was reported between Nephrology and Medicine wards [7], where admission to a closed-staff Nephology department resulted in a 20% mortality rate vs. a 52% mortality rate in the medical wards. Our study focused on 2 different cohorts of non-critically ill kidney patients and further extends the potential benefit of closed-staffed Nephrology care to all AKI stages and to CKD. Thus, we raise the hypothesis that a better outcome for hospitalized kidney patients is possible, not necessarily via advanced sophisticated technology, but rather involving hospital organizational steps promoting closed-staff Nephrology departments.

### Limitations

This study has some limitations. First, this is a single-center study and may not reflect other institutions. Nevertheless, since our single center is the only referral medical center in our region, this may be an advantage as missing data are unlikely. Second, the baseline characteristics of the Nephrology ward patients were more favorable as they were younger and had fewer cardiovascular comorbidities. In contrast, advanced CKD, and AKI stages, also known as major risk factors, were more prevalent in the Nephrology group. Although we used a robust propensity analysis to account for the difference in patients’ comorbidities, we cannot rule out an overlooked baseline risk factor that could have skewed our results. Third, we did not differ between hospitalization conferring bad prognosis, e.g., sepsis or MI, and hospitalization conferring a good prognosis, e.g., AV shunt surgery. Fourth, we don’t have the data regarding Nephology clinic visits after hospital discharge. However, this would seem to affect mostly the long-term outcomes rather than the short-term.

### Strengths

First, our more extensive study supports the results of a previous smaller study in a subgroup of AKI requiring acute dialysis [7] where admission to a Nephrology ward was also associated with reduced short-term mortality. Our study extends this finding to all stages of AKI and to CKD. Second, because missing data are unlikely in a single referral center, our study population appears to accurately reflect the kidney patient population in our area.

### Conclusion

Both AKI and CKD patients admitted to the Nephrology Department demonstrate significantly reduced short-term mortality, when compared to general Medicine departments. These findings highlight the human factor in kidney patient outcome, and support the role of highly trained, closed-staff Nephrology departments for specialized kidney patient care.

## Supporting information

S1 Table Exclusion criteria for CKD and AKI patients. (DOCX)

S1 Raw data (SAV)

S2 Raw data (XLSX)

S3 Raw data (SAV)

S4 Raw data (XLSX)
