# Supplementary material for: Admission of kidney patients to a closed staff nephrology department results in a better short-term survival
Source: PLoS One. 2023 Mar 7;18(3):e0279172. doi: 10.1371/journal.pone.0279172 (PMC9990939; doi:10.1371/journal.pone.0279172)
Supplement: S1 Table — (DOCX) [file pone.0279172.s001.docx]

**Table S1:** Exclusion criteria for CKD and AKI patients

| **AKI** | **CKD** | **Exclusion criteria** |
| --- | --- | --- |
| <18 y | < 18 y | **Age** |
| ICU or surgery ward | ICU or surgery ward | **Type of admission** |
| Excluded | Excluded | **Urgent dialysis on admission** |
| Excluded | Excluded | **ESRD** |
| Excluded | Excluded | **Absence of nephology consultation** |
| Excluded | Irrelevant | **Serum creatinine rise < 50% compared to baseline** |
| Irrelevant | Excluded | **eGFR> 60 ml/min** |
